# Therapeutic Effects of Tangshen Formula on Diabetic Nephropathy in *db/db* Mice Using Cytokine Antibody Array

**DOI:** 10.1155/2018/8237590

**Published:** 2018-02-22

**Authors:** Xue Mei Fan, Chun Lian Huang, Yi Ming Wang, Ning Li, Qiong Lin Liang, Guo An Luo

**Affiliations:** ^1^Beijing Key Laboratory of Bioorganic Phosphorus Chemistry & Chemical Biology (Ministry of Education), Department of Chemistry, Tsinghua University, Beijing, China; ^2^School of Pharmacy, China Pharmaceutical University, Nanjing, China; ^3^State Key Laboratory for Quality Research in Chinese Medicines, Macau University of Science and Technology, Macau, China

## Abstract

**Objective:**

Cytokines are essential promoters in the pathogenesis of diabetic nephropathy (DN) in type 2 diabetes. The following study investigates the adjustment mechanism of *Tangshen* formula (TSF) on cytokine expressions in *db/db* mice (DN animal model).

**Materials and Methods:**

*Db/db* mice were randomly divided into three groups. The treated groups were orally administered with TSF and losartan for 12 weeks. Biochemical and histological examinations were determined at 8 and 12 weeks posttreatment, while the cytokine antibody array analysis was applied to analyze the expression of 144 cytokines in kidney tissues at the end of the 12th week posttreatment.

**Results:**

TSF significantly reduced urinary albumin excretion and the levels of blood glucose, cholesterol, triglyceride, creatinine, and urea nitrogen. Furthermore, a significant decrease in glomerulus and mesangial area, as well as the downregulation of 24 cytokines and upregulated expressions of 5 cytokines, was found in the TSF-treated mice.

**Conclusions:**

The present study reveals that TSF could ameliorate the metabolic anomalies and renal injury in db/db mice. One of the important mechanisms for treatment of DN using the treatment of TSF is the control of the JAK/STAT signaling pathway via regulation of IL-2, IL-6, IL-13, Il-15, and IFN-*γ* expression.

## 1. Introduction

Diabetic nephropathy (DN) is one of the major microvascular complications in type 1 and type 2 diabetes and the leading cause of end-stage renal disease worldwide [1, 2]. Kidney inflammation has been reported to play an essential role in the development and progression of DN [3]. Many inflammatory cytokines are involved in the pathogenesis of DN, including cell adhesion molecules (CAMs), monocyte chemotactic protein-1 (MCP-1), tumor necrosis factor-*α* (TNF-*α*), interleukin-1 (IL-1), interleukin-6 (IL-6), interleukin-18 (IL-18), transforming growth factor-*β* (TGF-*β*), adiponectin, leptin, and resistin which are believed to be essential for DN because of cellular injury and progressive fibrosis [4]. In such circumstances, the multidetection of cytokines could be used to identify therapeutic mechanisms.

*Tangshen* formula (TSF), a traditional Chinese medicine composed of *Radix Astragali*, *Radix Rehmanniae*, *Radix Notoginseng*, *Radix et Rhizoma Rhei*, *Fructus Aurantii*, *Fructus Corni*, and *Ramulus Euonymi*, has been used to treat DN in clinic [5–7]. A recent clinical trial has proved the efficacy and safety of TSF in decreasing proteinuria and improving eGFR in patients with diabetic kidney disease and macroalbuminuria [5]. Previous studies have also shown that TSF is very important in regulating and ameliorating the metabolism of phospholipids [8] and purine-pyrimidine [9]. To date, there have been few reports about TSF regulating cytokine expression in patients with DN; thus, further investigations of the molecular mechanisms of TSF are needed. The purpose of this study was to evaluate the effects of TSF against DN and to explore the molecular mechanisms of multiple cytokine expressions underlying the renal-protective activity in *db/db* mice.

In addition, antihypertensive drugs such as angiotensin II-converting enzyme inhibitors (ACEI) or angiotensin II receptor blockers (ARBs) are commonly used to treat proteinuria in clinic. Losartan, angiotensin II receptor antagonists which are commonly used to treat high blood pressure, conferred significant renal benefits in patients with type 2 diabetes and nephropathy [10]. Thus, losartan was used as the positive control in this study.

## 2. Materials and Methods

### 2.1. Chemicals

Losartan potassium tablets (Lot: 110674, MSD, USA); *Tangshen* formula was provided by China-Japan Friendship Hospital (Lot: 0606320, Jiangyin Tianjiang Pharmaceutical, China); Albumin (Mouse) Elisa Kit (KA0489, Abnova, USA); RayBio®2 × Cell Lysis Buffer (Lot: 121,580, RayBiotech, USA); Biotechnologies BCA Protein Assay Kit (Lot: 201556AX, Aidlab, China); RayBio Mouse Cytokine Antibody Array G-Series 2000 (RayBiotech, USA).

### 2.2. Preparation of Tangshen Formula

TSF powder (prepared and standardized in Jiangyin Tianjiang Pharmaceutical, Jiangsu, China) was provided by China-Japan Friendship Hospital. Seven natural herbs, *Radix Astragali* (35.3%), *Radix Rehmanniae* (14.4%), *Radix Notoginseng* (3.5%), *Radix et Rhizoma Rhei* (7.1%), *Fructus Aurantii* (11.5%), *Fructus Corni* (10.6%), and *Ramulus Euonymi* (17.6%), were well mixed and soaked in distilled water for 30 minutes, boiled in 10 volumes of water (*v*/*w*) for 1 h, and extracted twice. The extract was filtrated and condensed to the final concentration of 1 g/mL and processed into powder by spray drying [11]. In our previous research, TSF had been subjected to high-performance liquid chromatography (HPLC) analysis; a Shimadzu diode array detector (DAD) was set at 280 nm to detect the constituents of TSF. Finally, fifty-nine compounds in *Tangshen* formula were identified, including flavonoids and flavonoid glycosides, iridoid glycosides, anthraquinone, and triterpenoid saponins [12].

### 2.3. Animals and Treatment Allocations

Obese diabetic mice lacking leptin receptor (*db/db*) carry a mutation in the leptin receptor gene and are well-established spontaneous models of obesity-induced type 2 diabetes. 19-week-old *db/db* male mice (C57BL/KsJ) weighing 40–60 g and *db/m* normal mice weighing 20–30 g were purchased from Vital River Laboratory Animal Technology Co. Ltd. (Beijing, China). All the animals were housed in an environment with temperature of 22 ± 1°C, relative humidity of 50 ± 1%, and a light/dark cycle of 12/12 hr. All animal studies (including the mouse euthanasia procedure) were done in compliance with the regulations and guidelines of Tsinghua University institutional animal care and were conducted according to the AAALAC and the IACUC guidelines (approval number: 12-LGA9; approval date: July 2012).

After acclimating to the laboratory for 1 week, biochemical indexes of blood taken from ocular venous plexus and urinary albumin in both *db/db* mice and *db/m* mice were measured, and the kidneys were collected from each mouse. Then the remaining mice were randomly divided into four groups (16 mice/group): (a) control group (*db/m* mice orally administrated with pure water); (b) model group (*db/db* mice orally administrated with pure water); (c) losartan group (*db/db* mice orally administrated with losartan, 6.50 mg/kg/day); (d) TSF group (*db/db* mice orally administrated with TSF, 2.08 g/kg/day). The treatment lasted for 12 weeks.

At 0, 8, and 12 weeks posttreatment, the mice were euthanized and the following experiments were performed: (1) blood was collected from ocular venous plexus and centrifuged at 4°C, 4000 rpm for 15 min, and the supernatant was further analyzed for biochemical indexes; (2) renal tissues were collected and immediately washed with cold phosphate-buffered saline (PBS); the left kidney was formaldehyde fixed for histological examination, while the right kidney was snap-frozen and stored in liquid nitrogen for cytokines detection.

Twenty-four-hour urine collections were obtained from the volume in each mouse after they were placed in metabolic cages the day before collecting blood samples; next, the urine was centrifuged at 4°C, 3000 rpm, for 15 min, and then the supernatant was taken to test the urinary albumin.

### 2.4. Blood and Urine Chemistry

The levels of blood glucose, cholesterol, triglyceride, blood urea nitrogen, and creatinine were measured by the Laboratory Medicine, Peking University Hospital. Urinary albumin was strictly measured by Albumin (Mouse) Elisa Kit according to manufacturer's instructions. The urinary albumin excretion was expressed by the total amount excreted in 24 h.

### 2.5. Histological Examination

Formaldehyde-fixed kidney tissues were embedded in paraffin. Sections of 4 *μ*m thickness were cut and stained with hematoxylin and eosin (HE) and periodic acid–Schiff base (PAS). The pathological changes were observed under light microscope. PAS staining photographs were obtained and quantitatively analyzed with SPI analysis software for morphology. About 10 glomeruli from each slide were randomly selected and checked by a computed image analyzer. The mean glomerular area, mesangial area, and the mesangial area to glomerular area ratio were calculated. The percent of the mesangial area in the glomerular area in preparations stained with PAS was used as a glomerular sclerotic index. Differences in the areas and ratios mentioned above between different groups were analyzed by Student's *t*-test.

### 2.6. Detection of Renal Cytokines by Cytokine Antibody Array

Right kidneys from the model group in expt. week 0 and the right kidneys from model, TSF, and losartan groups in expt. week 12 then were ground under liquid nitrogen with a prechilled mortar and pestle and homogenized and treated with lysis buffer (50 mmol/L Tris, pH 7.4, 150 mmol/L NaCl, 1% Triton X-100, 1% sodium deoxycholate, 0.1% SDS, sodium orthovanadate, sodium fluoride, EDTA, etc.). The total protein concentration was analyzed using BCA protein assay kit according to the manufacturer's instructions. Each extract was diluted to the same protein concentration (1 *μ*g/*μ*L) before the cytokine antibody array analysis was performed.

Renal cytokine levels of mice were analyzed using Mouse Cytokine Antibody Array G-Series 2000 from RayBiotech, containing 144 different anti-cytokine antibodies, as well as positive and negative controls (http://www.raybiotech.com/files/manual/Antibody-Array/AAM-CYT-G2000.pdf). The assays were strictly performed according to the manufacturer's instructions. The glass slides were scanned with CapitalBio LuxScan 10 K-A Microarray Scanner using Cy3 channel. Fluorescence (intensities of obtained spots) was recorded to determine the changes in detected cytokine expressions.

### 2.7. Microarray Data Extraction and Analysis

The cytokine expression data were imported into the mouse cytokine semiquantitative analysis software (S02-AAM-CYT-G2000) for correction and normalization. The Matlab software (version 5.0) was used for further cluster analysis. The pathway analysis was performed on DAVID and Kyoto Encyclopedia of Genes and Genomes (KEGG) pathways.

### 2.8. Statistical Analysis

Values are expressed as means ± SEM, with *n* denoting the number of animals. Significance was evaluated using one-way ANOVA. Comparisons between two groups were performed using Student's *t*-test. *P* < 0.05 was considered statistically significant, while *P* < 0.01 was considered highly statistically significant.

## 3. Results

### 3.1. TSF Treatment Inhibits Blood Glucose Increase and Regulates the Disorder of Glycolipid Metabolism in *db/db* Mice

The levels of blood glucose, triglyceride, and cholesterol were measured to reveal the adjustment of TSF on the glycolipid metabolism in *db/db* mice (Tables 1 and 2). Briefly, the levels of blood glucose, triglyceride, and cholesterol in model group were significantly elevated compared to those of the control group. After 8 and 12 weeks of treatment, TSF administration groups showed a tendency to decrease the abnormal levels of glucose, triglyceride, and cholesterol. Nevertheless, no significant differences in cholesterol levels were observed in the in losartan-treated group compared to the model group. To sum up, these results indicated that TSF might effectively control the blood glucose and regulate glycolipid metabolism disorder.

### 3.2. TSF Treatment Attenuates Renal Dysfunctions

#### 3.2.1. TSF Treatment Alleviates the Renal Dysfunctions in *db/db* Mice

The levels of urinary albumin excretion, blood creatinine, and urea nitrogen were detected to evaluate the diabetic renal dysfunction. As shown in Table 2, the levels of blood creatinine, urea nitrogen, and urinary albumin were significantly higher in the model group compared to the control group, while they were efficiently reduced in mice treated with TSF and losartan. This data suggested that TSF alleviates the renal dysfunctions in *db/db* mice.

#### 3.2.2. TSF Treatment Decreases Kidney Tissue Damage in *db/db* Mice

**Figure 1:**
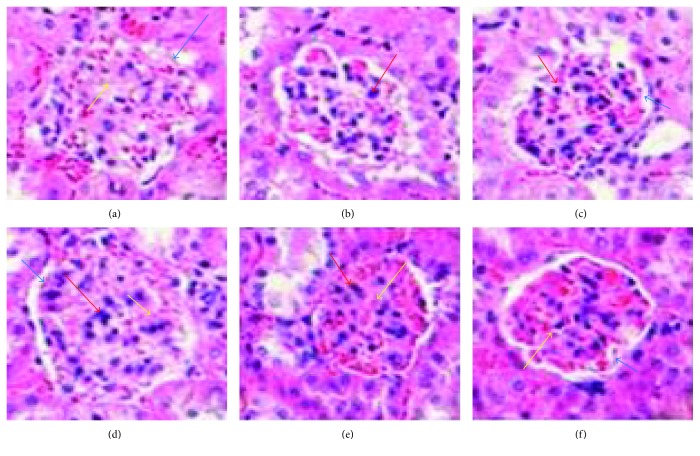
Renal pathological changes after 12-week treatment (HE × 400). (a) Model group (expt. week 0). (b) Control group (expt. week 0). (c) Control group (expt. week 12). (d) Model group (expt. week 12). (e) Losartan group (expt. week 12). (f) TSF group (expt. week 12). The red arrow indicates mesangial cells, the yellow arrow indicates endothelial cells, and the blue indicates basement membrane.

The HE staining results of kidney tissues are shown in Figure 1. After 12 weeks, *db/m* kidney tissues had intact glomerular morphology, clean cut, regular arrangement, and normal tubular structure. Contrary, increased glomerular volume, endothelial and mesangial cell proliferation, narrow capsular space, and blurred tubular structure were observed in the model group. In TSF group, mesangial expansion and glomerular volume were markedly ameliorated and the tubular structure was clear compared with the mouse model group of the same age. Similar improvement was observed in the losartan group.

**Figure 2:**
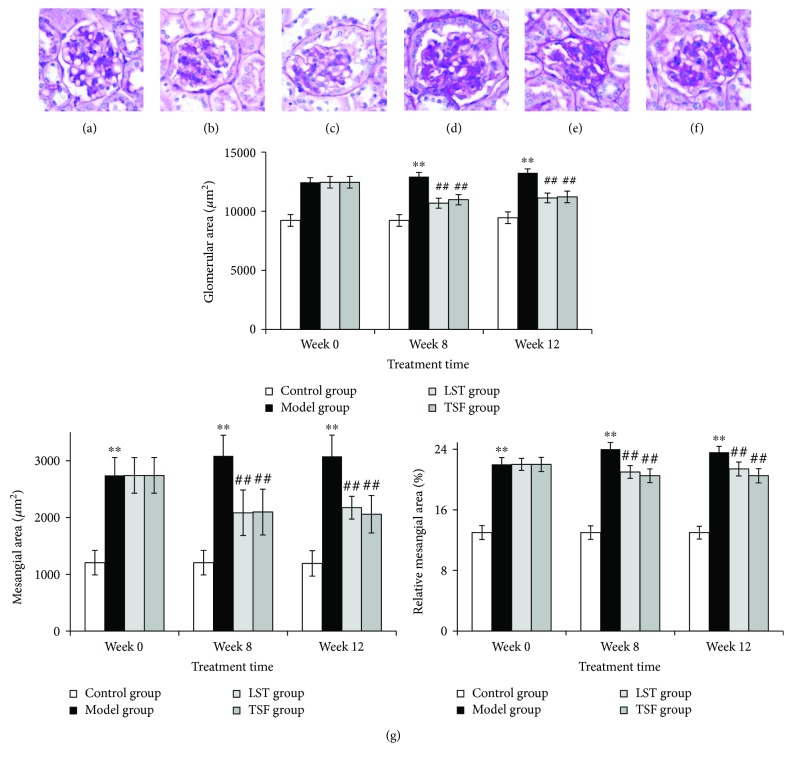
Renal pathological changes after 12-week treatment (PAS × 400). (a) Model group (expt. week 0); (b) control group (expt. week 0); (c) control group (expt. week 12); (d) model group (expt. week 12); (e) LST (losartan) group (expt. week 12); (f) TSF group (expt. week 12); (g) change of glomerular area, mesangial area, and the percentage of mesangial area. ^∗∗^*P* < 0.01 compared with control group in the same time point; ^##^*P* < 0.01 compared with the model group at the same time point.

According to the quantitative analysis results of PAS staining, glomerular fibrotic areas were significantly increased in *db/db* mice compared to the control group (Figure 2). After 12-week treatment with TSF, there was a significant decrease in the glomerular surface area, as well as in mesangial matrix and relative mesangial matrix area compared to model mice; while no significant differences were observed between the treated groups (Figure 2(g)).

#### 3.2.3. TSF Treatment Improves the Abnormal Expression of Cytokines in Kidney Tissue of *db/db* Mice

We further examined the therapeutic effect of TSF on 144 cytokine expression in plasma of *db/db* mice using cytokine antibody array. After the semiquantitative soft analysis of the mouse cytokines, a strict significant *t*-test resulted in a final set of 29 differentially expressed cytokines, including 24 cytokines that were downregulated and 5 that were upregulated in *db/db* mice after TSF treatment. 29 cytokines and the changes in their expression are shown in Table 3.

**Figure 3:**
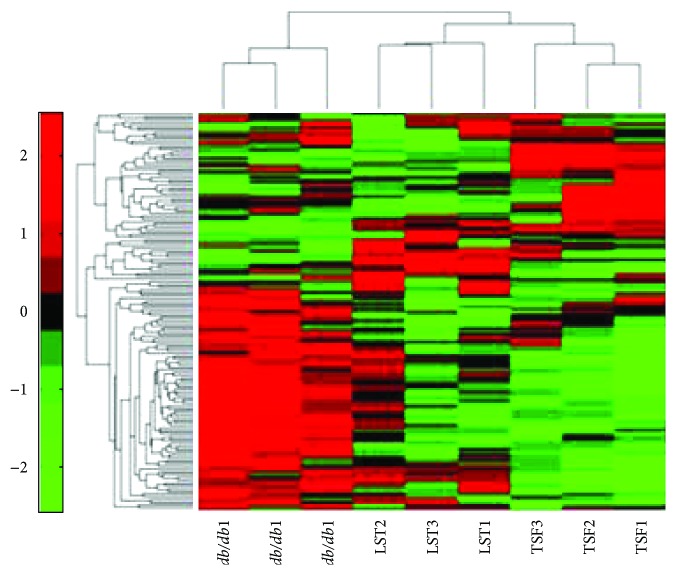
Cluster analysis result of protein microarray data. Model group, LST (losartan) group, and TSF group have 3 samples which represent biologically independent duplicates. Red indicates increased expression; green indicates reduced expression.

Cluster analysis was applied to observe the overall expression changes of cytokines among model, TSF and losartan groups. Before cluster analysis, the data of expt. week 12 was normalized with the data of expt. week 0's *db/db* mice as a reference. Then cluster result of microarray data was generated by Matlab 5.0 (Figure 3). The TSF and losartan groups could get together for a class and all are well separated separated with the model group. The results also showed that changed cytokines of the TSF group were farther away from the model group than from losartan group, indicating that TSF has a unique and effective molecular regulatory mechanism in the treatment of DN.

#### 3.2.4. Regulated JAK/STAT Signaling Pathway Regulated via Improved the Related Cytokine Abnormal Expression May Be Mechanisms by TSF Treatment DN in *db/db* Mice

**Figure 4:**
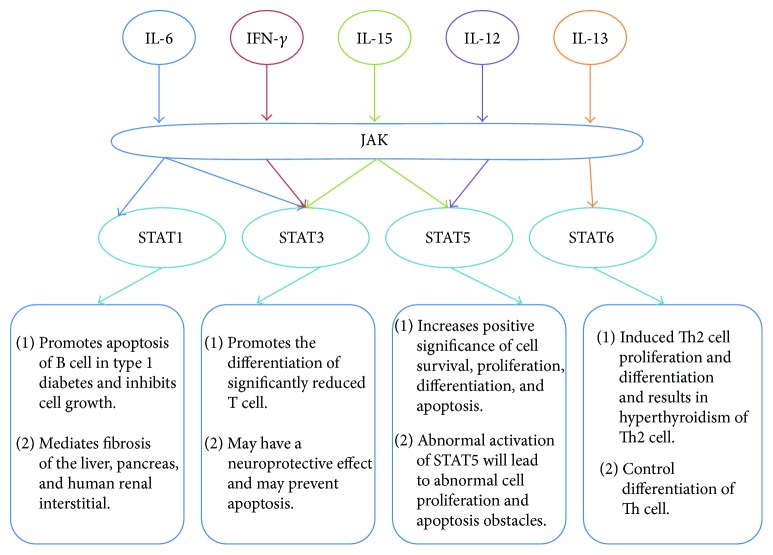
The main physiological and pathological role of IL-2, IL-6, IL-13, IL-15, and IFN-*γ*.

To gain insights into the biological roles of the differentially expressed cytokines adjusted by TSF, the pathway enrichment analysis was performed on DAVID software (KEGG pathway analysis). The significant pathways (*P* < 0.001) were cytokine-cytokine receptor interaction, JAK/STAT signaling pathway, adipocytokine signaling pathway, Toll-like receptor signaling pathway, and cell adhesion molecules (CAMs). Cytokine-cytokine receptor interaction and JAK/STAT signaling pathway were the most significant channels. In the cytokine-cytokine receptor interaction, the cytokines with altered expression were widely distributed. In the JAK/STAT signaling pathway, the distribution of differentially expressed cytokines was more concentrated. Moreover, JAK/STAT pathway might be closely related to the pathogenesis of DN [13, 14]. Therefore, we made a further investigation of these 5 differentially expressed cytokines (Table 4) in JAK/STAT signaling pathway related to DN.  Figure 4 showed the main role of these cytokines and related JAK/STAT signaling pathway in physiology and pathology.

## 4. Discussion

DN is one of the major microvascular complications in type 2 diabetes and a leading cause of end-stage renal disease. Using traditional Chinese medicine for DN has shown good results and has become increasingly recognized worldwide. In the present study, we verified the efficacy of TSF and discussed its action mechanisms on animal models of DN.

The determination of biochemical indexes indicated that TSF could effectively control the deterioration of DN. The levels of blood glucose, blood triglyceride, and cholesterol significantly decreased following TSF treatment; these beneficial effects can be achieved via TSF regulating action on the glycolipid metabolism. Urinary albumin, blood urea nitrogen, and blood creatinine are commonly used to characterize the renal dysfunction in clinical diagnosis. As shown in Tables 1 and 2, the urinary albumin, blood urea nitrogen, and blood creatinine levels were all reduced with TSF treatment, suggesting that TSF could effectively reduce kidney damage caused by DN. In pathological analysis, treatment with TSF resulted in a significant reduction in formation of glomerular sclerotic lesions and fibrosis that were marked in the model group. These observations suggested that the intake of TSF could slow down the progressive deterioration in pathological aspects of DN in *db/db* mice.

Above-mentioned results showed that TSF therapy could indeed slow down the disease progression of DN. On this basis, the molecular mechanism of TSF was further explored by cytokine antibody array. The results of protein microarray analysis revealed that the occurrence and development of DN were regulated by a variety of cytokines and growth factors. In this study, the expression of 29 cytokines (in total 144 proteins) significantly changed compared with the model group after TSF intervention, including IL-2, IL-6, IL-13, IL-15, and IFN-*γ* enriched to JAK/STAT signaling pathway. JAK/STAT pathway, an essential intracellular mechanism of cytokines and growth factors, is frequently associated with gene expression and cellular activation, proliferation, and differentiation in DN [13, 14]. In this animal experiment, the genomics of renal tissue was carried out in parallel. And the results have provided evidence for the involvement of JAK/STAT/SOCS pathway in the mechanism of Tangshen formula-treated diabetic nephropathy at the gene expression level [12]. Growth factor and inflammatory factor signaling pathways mediated by JAK/STAT are considered to be very important in diabetic nephropathy [15]. When experimental time was prolonged, the IL-2, IL-6, IL-13, and IL-15 expressions and IFN-*γ* were abnormally increased in the model group. However, decreased levels of these five cytokines implied that JAK/STAT pathway was inhibited following the TSE treatment. This means that TSF may affect the downstream function by regulating the expression of the five cytokines.

IL-2 has a very complex role in the development and control of inflammatory disease [16]. Accordingly, major IL-2 dysfunctions in both humans and mice are associated with the development of autoimmunity as well as immunodeficiencies [17, 18]. This finding highlights that the balance between the IL-2 pro- and anti-inflammatory effects is critical for an appropriate mounting and resolution of immune responses. However, there are no previous studies that have revealed the functionality of IL-2 in kidney tissue diagnosis of DN. In our study, renal expression of IL-2 was decreased in TSF-treated *db/db* mice compared with the model group. This suggested that TSF could adjust the renal level of IL-2 to an appropriate standard to improve renal immunity in *db/db* mice, eventually reducing the kidney damage.

IL-6 is a well-known pleiotropic cytokine which is the classic upstream activator of the JAK/STAT3. It has been shown that inhibition of the JAK/STAT3 pathway reduced infiltration of interstitial inflammatory cells and production of chemokines in the adriamycin-induced nephropathy model [19]. IL-6 levels and polymorphisms were also associated with the risk of DN [20]. It was reported that IL-6 was associated with reduced glomerular filtration rate (GFR) in type 2 diabetic patients [21]. Choudhary and Ahlawat have also confirmed that the elevated levels of IL-6 are independently associated with urinary albumin excretion [22]. *Lu Huang Shen Hua* capsule, a traditional Chinese medicine, could reduce urine albumin excretion, lower serum IL-6, and TNF-*α* levels by controlling FBG, thus having an important role in kidney protection and DNA prevention and treatment [23]. In the present study, decreased levels of IL-6 in renal and lower albuminuria were observed in TSF group compared with the model group. Therefore, we believe that TSF could attenuate the albuminuria by indirectly decreasing the IL-6 expression in renal.

IL-13 triggers a number of signaling pathways, and different effects of IL-13 are mediated through discrete mechanisms. IL-13 that activates JAK3/STAT6 signaling, including the kidney [24], has been identified as the principle signaling pathway involved in IL-13-induced responses [25]. The previous study has suggested that IL-13 overexpression in the rat could lead to podocyte injury with downregulation of nephrin, podocin, and dystroglycan, and a concurrent upregulation of B7-1 in the glomeruli, inducing a minimal change-like nephropathy that is characterized by increased proteinuria, hypoalbuminemia, hypercholesterolemia, and fusion of podocyte foot processes [26]. IL-13 has a pathogenic role in altered glomerular permeability by influencing intracellular trafficking of proteins and promoting proteolysis at the basolateral surface of glomerular visceral epithelial cells [27]. TSF treatment can significantly reduce the levels of kidney IL-13 in renal and the urine albumin excretion; therefore, we speculate that TSF may protect the renal function by regulating the IL-13 renal levels in *db/db* mice.

IL-15 is an autocrine survival factor for tubular epithelial cells, both in humans and mice that has a vital role in renal physiology [28, 29]. Increased or decreased expression of IL-15 characterizes several renal pathologies that are harmful to renal cell survival and kidney functions [30]. On the other hand, the synthesis of increased IL-15 has been linked with autoimmune diseases, such as diabetes mellitus type 1 and 2 [31]. Indeed, we found that IL-15 level in renal mouse model was significantly increased compared with the TSF-treated mice, suggesting that increased expression of IL-15 may enhance proinflammatory actions leading to the renal fibrosis. TSF could reduce the levels of IL-15 in kidney, thus ameliorating the inflammation in *db/db* mice. This finding was consistent with the supposition that although high IL-15 level enhances proinflammatory actions, extremely low IL-15 level markedly and selectively suppresses proinflammatory actions [32].

IFN-*γ* induces gene transcription via activation of the JAK/STAT pathway [33]. Inflammation modulates angiotensinogen (AGT) production in tissues via inflammatory cytokines. Previous studies have shown that IFN-*γ* increases AGT expression in hepatocytes via activating the STAT1 pathway [34], while Satou et al. have reported that IFN-*γ* radically regulates AGT expression in the kidney via STAT3 activity modulated by STAT1-SOCS1 axis [35]. In the present study, IFN-*γ* expression in the kidney of the TSF group was significantly attenuated, indicating that TSF could reduce AGT production by reducing the expression of IFN-*γ* and thus ameliorate the development of hypertension. However, the exact way of how the TSF regulates the expression of IFN-*γ* needs to be further investigated.

## 5. Conclusions

This study demonstrated that TSF has the ability to ameliorate impaired metabolic status of blood glucose and lipid, to improve renal function, and to slow down the renal fibrosis by regulating the JAK/STAT pathway. The next stage is to make further analysis of differentially expressed cytokines.

## Figures and Tables

**Table 1:** Blood and urine chemistry values in *db/db* mouse groups, 8 weeks posttreatment. (*n* = 8, means ± SEM).

Index	Control group	Model group	Losartan group	TSF group
Glucose (mmol·L^−1^)	6.01 ± 0.24	26.53 ± 5.57^∗^	15.60 ± 4.73^#^	13.80 ± 3.95^#^
Triglycerides (mmol·L^−1^)	0.70 ± 0.02	0.96 ± 0.10^∗^	0.69 ± 0.05^#^	0.73 ± 0.04^#^
Cholesterol (mmol·L^−1^)	2.34 ± 0.09	4.77 ± 0.46^∗^	3.80 ± 0.56	3.66 ± 0.31^#^
Creatinine (*μ*mol·L^−1^)	35.23 ± 5.48	42.67 ± 5.49^∗^	44.20 ± 7.00	35.67 ± 2.93^#^
Blood urea nitrogen (mmol·L^−1^)	6.64 ± 0.36	9.38 ± 0.32^∗^	7.94 ± 0.31^#^	7.40 ± 0.58^#^
Urinary albumin (*μ*g/24 h)	28.6 ± 2.60	288.00 ± 21.91^∗^	267.00 ± 17.96	273.00 ± 18.78

^∗^ *P* < 0.05 compared with the control group. ^#^*P* < 0.05 compared with the model group.

**Table 2:** Blood and urine chemistry values in *db/db* mouse groups, 12 weeks posttreatment. (*n* = 8, means ± SEM).

Index	Control group	Model group	Losartan group	TSF group
Glucose (mmol·L^−1^)	7.08 ± 0.64	34.00 ± 3.48^∗^	14.72 ± 2.77^#^	14.91 ± 2.94^#^
Triglycerides (mmol·L^−1^)	0.64 ± 0.03	1.13 ± 0.04^∗^	0.96 ± 0.07^#^	0.95 ± 0.05^#^
Cholesterol (mmol·L^−1^)	2.51 ± 0.11	4.63 ± 0.27^∗^	4.26 ± 0.26	4.07 ± 0.15^#^
Creatinine (*μ*mol·L^−1^)	35.50 ± 2.56	44.86 ± 3.07^∗^	30.86 ± 4.13^#^	32.22 ± 4.14^#^
Blood urea nitrogen (mmol·L^−1^)	6.05 ± 0.39	7.28 ± 0.33^∗^	6.36 ± 0.58^#^	6.05 ± 0.39^#^
Urinary albumin (*μ*g/24 h)	32.80 ± 2.37	314.00 ± 19.65^∗^	225.00 ± 12.73^#^	240.00 ± 12.00^#^

^∗^ *P* < 0.05 compared with the control group. ^#^*P* < 0.05 compared with the model group.

**Table 3:** The relative expression changes of 29 cytokines.

Number	Cytokines	Model group (expt. week 12)/model group (expt. week 0)	LST group (expt. week 12)/model group (expt. week 0)	TSF group (expt. week 12)/model group (expt. week 0)
1	VEGF	0.94	0.97	0.75
2	VEGF R2	0.90	1.48	0.64
3	IGFBP-3	1.13	1.05	0.96
4	SCF	0.95	1.00	0.67
5	IL-2	1.52	1.47	1.01
6	IL-4	3.72	3.73	2.02
7	IL-6	1.61	1.26	1.32
8	IL-13	1.19	1.14	0.96
9	IL-15	1.05	0.82	0.83
10	FAS ligand	0.99	0.78	0.65
11	ICAM-1	0.54	0.69	0.42
12	VCAM-1	0.35	0.42	0.24
13	PF4	0.93	0.67	0.56
14	TIMP-1	0.99	1.05	0.73
15	IFN-gamma	1.76	1.70	1.51
16	Decorin	2.02	1.88	1.56
17	TNF-alpha	1.50	1.26	1.27
18	IL-3	0.76	0.68	0.51
19	IL-7	1.37	1.18	1.01
20	IL-10	0.74	0.63	0.59
21	IL-17	0.74	0.65	0.54
22	MCP-1	0.64	0.59	0.41
23	M-CSF	0.70	0.64	0.53
24	CD36	0.74	0.63	0.49
25	EGF	0.59	0.36	0.75
26	IGFBP-2	0.74	0.65	0.98
27	IGF-II	0.64	0.59	0.86
28	MMP-2	0.70	0.64	0.95
29	E-cadherin	1.05	1.01	1.23

**Table 4:** Semiquantitative results of 5 differentially expressed cytokines in the JAK/STAT signaling pathway (means ± SEM; 3 individuals).

Cytokines	Fluorescence intensity value (IU)			
	Expt. week 0 Model group	Expt. week 12 Model group	Expt. week 12 Losartan group	Expt. week 12 TSF group
IL-2	3840 ± 83	5846 ± 202	5626 ± 94	4628 ± 116^∗^
IL-6	1226 ± 42	1970 ± 68	1544 ± 55^∗^	1622 ± 55^∗^
IL-13	1020 ± 37	1210 ± 29	1161 ± 42	974 ± 23^∗^
IL-15	2163 ± 71	2771 ± 104	2156 ± 101^∗^	2196 ± 116^∗^
IFN-*γ*	1630 ± 55	2861 ± 25	2768 ± 89	2461 ± 103^∗^

^∗^ *P* < 0.05 compared with the model group in expt. week 12.
